# The Protective Effect of Polysaccharide SAFP from *Sarcodon aspratus* on Water Immersion and Restraint Stress-Induced Gastric Ulcer and Modulatory Effects on Gut Microbiota Dysbiosis

**DOI:** 10.3390/foods11111567

**Published:** 2022-05-26

**Authors:** Dongjing Zhang, Ming Xiang, Yun Jiang, Fen Wu, Huaqun Chen, Min Sun, Lingzhi Zhang, Xianfeng Du, Lei Chen

**Affiliations:** 1 Anhui Key Laboratory of Eco-Engineering and Biotechnology, School of Life Sciences, Anhui University, Hefei 230601, China; zhangdongjing1987@163.com (D.Z.); ahxiangming@163.com (M.X.); jiangyun725@126.com (Y.J.); wufen2021@163.com (F.W.); 19105607612m@sina.cn (H.C.); sunmin@ahu.edu.cn (M.S.); 2 School of Biological and Food Engineering, Suzhou University, Suzhou 234000, China; 3 Anhui Cordyceps Source Biotechnology Co., Ltd., Huainan 232000, China; ah_ccy@163.com; 4 State Key Laboratory of Tea Plant Biology and Utilization, School of Tea and Food Science & Technology, Anhui Agricultural University, Hefei 230036, China

**Keywords:** *Sarcodon aspratus* polysaccharides, WIRS-induced gastric ulcer, gut microbiota, signaling pathway

## Abstract

*Sarcodon aspratus* is a popular edible fungus for its tasty flavour and can be used as a dietary supplement for its functional substances. This study was conducted to evaluate the potential health benefits of *Sarcodon aspratus* polysaccharides (SAFP) on water immersion and restraint stress (WIRS)-induced gastric ulcer in rats. The results indicated that SAFP could decrease myeloperoxidase (MPO) activity and plasma corticosterone levels, as well as enhance Prostaglandin E2 (PGE2) and Nitrate/nitrite (NOx) concentration in rats. Furthermore, SAFP significantly attenuated the stress damage, inflammation, pathological changes and gastric mucosal lesion in rats. Moreover, high-throughput pyrosequencing of 16S rRNA suggested that SAFP modulated the dysbiosis of gut microbiota by enhancing the relative abundance of probiotics, decreasing WIRS-triggered bacteria proliferation. In summary, these results provided the evidence that SAFP exerted a beneficial effect on a WIRS-induced gastric ulcer via blocking the TLR4 signaling pathway and activating the Nrf2 signaling pathway. Notably, SAFP could modulate the WIRS-induced dysbiosis of gut microbiota. Thus, SAFP might be explored as a natural gastric mucosal protective agent in the prevention of gastric ulcers and other related diseases in the food and pharmaceutical industries.

## 1. Introduction

As one of the most common diseases of the digestive system worldwide, gastric ulcer (GU) can be triggered by a variety of factors, including unhealthy eating habits, helicobacter pylori infection, excessive alcohol consumption, use of nonsteroidal anti-inflammatory drugs (NSAIDs) and destruction of the gastric mucosal protective barrier [1,2]. Stress gastric ulcer is an acute haemorrhagic injury characterised by ulceration of the gastric mucosa caused by environmental stress, the decline in the mucosal prostaglandin and mucoprotein, an increase in oxidative stress and neutrophil activation and the suppression of the gastric mucosal cell proliferation [3,4]. Currently, the continued use of peptic ulcer drugs such as histamine-H-2 receptor antagonists and proton pump inhibitors for a long time could result in a high GU recurrence rate and poor healing quality, even gut microbiota dysbiosis [5,6], and bring about inevitable side effects such as arrhythmias, hypomagnesaemia, allergies, impotence and gynaecological mastitis [7]. Therefore, it has already become a current research focus to develop some natural, safe and effective gastro protective agents from food sources with lower toxicity and fewer side effects.

Recently, there has been growing evidence that gut microbiota is one of the key factors that could affect nutrient metabolism and immune response, to maintain the health of the host when faced with a variety of intestinal diseases [8]. Several studies have demonstrated that intestinal microbiome disorders are closely related to autoimmune and gastrointestinal diseases such as GUs, inflammatory bowel disease (IBD) and metabolic syndrome [9,10]. The gut microbiota greatly delay the progression of gastrointestinal diseases by regulating the intestinal endothelial barrier function [11], promoting the regeneration of vascular endothelial factors and healing of gastric mucosa, chronic inflammation caused by metabolic endotoxemia [12] and immune activation and playing an important role in maintaining metabolism homeostasis. Therefore, reshaping the intestinal microbiota by diet, such as functional food, or other means has been proven to have beneficial effects in maintaining the integrity of intestinal function and relieving gastric mucosal damage [13].

Polysaccharides are the main bioactive compounds of mushrooms, which have attracted extensive attention in academia due to their functional properties and non-toxic side effects, such as glucose and lipid metabolism regulation [14], antioxidant functions [15], anti-inflammatory [16] and immunostimulating effects [17]. There is a growing interest in the therapeutic and prophylactic effects of polysaccharides by modulating intestinal microbiota and metabolic syndrome [12,18]. Some researchers have demonstrated that polysaccharides were used as an alternative therapy in several inflammatory diseases, such as GUs, chronic gastritis, ulcerative colitis and IBD, by activating antioxidant defences [16,19]. *Sargassum fusiforme* polysaccharides exhibited significant anti-inflammatory and antioxidant properties against ethanol-induced GUs by altering various molecules, such as nuclear factor-kappa B, IκB, nitrotyrosine and cyclooxygenase-2 [20]. The gastroprotective activity of the polysaccharide fraction from *Bletilla* *striata* could be attributed to the reduction of pro-inflammatory cytokines and oxidative stress by the inhibition of MAPK/nuclear factor-κB (NF-κB) pathways [21].

*Sarcodon aspratus*, as a kind of popular wild edible fungi, mainly grows in Yunnan province, Sichuan province and the Xinjiang uygur autonomous region of China. Sarcodon aspratus has commonly been used as a functional food in China and other Asian countries for its taste and health values, with non-toxic side effects. *Sarcodon aspratus* is very delicious and nutritious, and rich in protein, carbohydrates and a variety of minerals [22]. Polysaccharides from *Sarcodon aspratus* were usually extracted by the method of water extract and alcohol precipitate, followed by protein removal using the Sevag method [23]. It has been proven that polysaccharides extracted from *Sarcodon aspratus* can markedly reduce HFD-induced body weight gain and fat accumulation and improve lipid homeostasis and glucose tolerance in HFD-fed mice [14]. Our previous studies also indicated that *Sarcodon aspratus* polysaccharides can alleviate oxidative stress-induced cell damage and bleomycin-induced pulmonary fibrosis [22]. However, to our best knowledge, it remains unknown whether *Sarcodon aspratus* fruiting polysaccharides (SAFP) have any beneficial effect on GUs in a rat model, and the regulating effect of SAFP on intestinal flora dysbiosis has not been studied. Thus, in this study, we aimed to evaluate the protective effect of SAFP on water immersion and restraint stress (WIRS)-induced gastric mucosal injury in SD (Sprague-Dawley) rats. In addition, the favourable effects of SAFP on intestinal flora homeostasis in GU rats were also investigated.

## 2. Materials and Methods

### 2.1. Materials

The *Sarcodon aspratus* fruiting polysaccharides (SAFP) were extracted from the dry fruiting bodies of *Sarcodon aspratus* (Yimeng Yisheng edible fungus cooperative, Yunnan, China) according to the method described in the previous study [23]. Our previous study found that the polysaccharide content was 86.75% and it was mainly composed of D-mannnose, D-glucose, D-galactose and L-fucose with molar ratios of 1.0:5.16:4.75:1.34 [22]. All reagents were of an analytical grade or higher and purchased from Runji Chemical Reagent Business Department (Hefei, China).

### 2.2. Intrinsic Viscosity

Polysaccharides SAFP were dissolved at 5–80 mg/mL in deionised water. The solutions were placed in a sealed test tube and left overnight to ensure complete solubilisation. Steady shear viscosities of SAFP solutions at various mass concentrations (5, 10, 20, 40, 80 mg/mL) were measured on a rotational rheometer (MCR 302, Anton Paar, Graz, Austria) at 25 °C with a shear rate regarded as independent variables with a range used from 0.01 to 1000 s^−1^.

Polysaccharides SAFP were dissolved at 20 mg/mL in deionised water. The solutions were placed in a sealed test tube and left overnight. Steady shear viscosities of 20 mg/mL SAFP solutions at different temperaturse (5, 15, 25, 35, 45, 55, 65, 75 °C) were measured on a rotational rheometer (MCR 302, Anton Paar, Graz, Austria) with a shear rate from 0.01 to 1000 s^−1^.

### 2.3. Animal Experiment

After forty-eight Sprague-Dawley (SD) rats (200 ± 16 g, 6-week-old) were adapted to feeding for 1 week, rats were randomly divided into six groups of 8 rats: the control group; stress gastric ulcer model group (SGUM group); SAFP groups including the SAFP low-dose group (SAFPL group), SAFP middle-dose group (SAFPM group) and SAFP high-dose group (SAFPH group); and Omeprazole group (OME group). After preliminary experiments to determine the dosages of SAFP and Omeprazole, rats were gavaged with SAFP (100, 200, 400 mg/kg BW) in the SAFP group and Omeprazole (30 mg/kg) in the OME group, respectively. The other rats were gavaged with normal saline (50 μL) instead. All rats were administered once a day for 15 consecutive days.

Then, all rats, except the control group, were fasted for 24 h with free access to drinking water before modelling. The conscious rats were restrained individually in rectangular polypropylene cages and immersed up to the depth of the xiphoid process in a temperature-controlled water bath (23 °C) for 10 h to induce WIRS as described previously [24]. After completing the experiment, all rats were sacrificed under chloral hydrate anaesthesia (10%, 0.35 mL/100 g), and the experiment was terminated after sampling. Their blood was taken from the abdominal aorta and centrifuged at 1500 g for 20 min to obtain plasma. To confirm that the stress model was generated correctly, plasma myeloperoxidase activity and plasma corticosterone levels were measured using a commercially available enzyme immunoassay kit (Nanjing Jiancheng Bioengineering Institute, Nanjing, China). After stripping the fat from other abdominal organs, the stomach was exposed and ligated at the pylorus. Then, 5 mL of 4% paraformaldehyde solution was injected into the stomach through the duodenum and the pylorus. The stomach was fixed for 10 min. Next, the stomach was cut open and flattened along the greater curvature to expose the gastric contents on the surface of the gastric mucosa. Then, they were gently rinsed with normal saline. The gastric tissues were flattened to observe the mucosal damage and ulcer morphology with a magnifying glass with a straight handle. In all gastric tissues, the total length (mm) of each linear haemorrhagic erosion was measured as the ulcer index (UI) (mm). All serum and tissue samples were frozen in liquid nitrogen and kept at a temperature of 80 °C until they were analysed. All the experimental procedures were approved by the Laboratory Animal Welfare and Ethics Committee of Anhui University (NO:2020-003), and conducted according to the Guide for the Care and Use of Laboratory Animals of the People’s Republic of China (GB/T35892-2018).

### 2.4. Determination of Biochemical Indicators in Serum and Gastric Tissues

One portion of the gastric tissues was homogenised in Tris-buffer on ice and centrifuged for 15 min at 3000× *g* rpm to obtain the supernatant for the determination of the levels of nitrate/nitrite and PGE-2. The nitrate/nitrite concentration in the gastric tissues was determined by using a colorimetric assay kit (Cayman, Ann Arbor, MI, USA, 780001), and the concentration was expressed in moles per milligram of protein. The prostaglandin E2 (PGE2) assay was carried out with the PGE2 Enzyme Immunoassay Kit (Cayman, Ann Arbor, MI, USA, 514010) and the PGE2 level in the gastric mucosa is expressed as picograms per milligram of tissue. In addition, the levels of SOD, MDA and GSH-Px were also determined according to the kit’s instructions. The other portion of gastric tissues homogenate was centrifuged for 15 min at 5000× *g* rpm to determine the level of inflammatory cytokines (TNF-α, IL-1β and IL-6) according to the kit’s instructions.

### 2.5. Histopathological Analysis

The gastric tissues were preserved by perfusion fixation with a solution of 4% paraformaldehyde, followed by dehydration and embedding in paraffin. Afterwards, the gastric tissues were stained with haematoxylin and eosin by using standard techniques. The morphological changes of gastric tissues were observed by an electron microscope (Olympus-IX73-DP80; Olympus Corp., Tokyo, Japan).

### 2.6. Immunohistochemistry Studies

The paraffin-embedded gastric tissues were dewaxed, rinsed with phosphate-buffered saline (PBS, pH 7.4) three times for 5 min and treated with 10% FBS at 37 °C for 30 min. Sequentially, the gastric tissues were dewaxed with dimethyl benzene and an ethanol gradient, washed with phosphate-buffered saline (PBS) for 3 min each time for a total of three times and treated with 10% FBS at 37 °C for 30 min. Next, sections were incubated with primary antibodies against Keap1 (1:500, ab196346, Abcam, Cambridge, UK), Nrf2 (1:200, ab137550, Abcam, Cambridge, UK), TLR4 (1:500, ab22048; Abcam, Cambridge, UK) and NF-κB (1:500, ab16502; Abcam, Cambridge, UK) in a humidified chamber at 4 °C overnight. The sections were incubated with HRP-labelled secondary antibodies for 30 min after being washed with PBS three times. Then, 3,3′-diaminobenzidine (DAB, Sigma-Aldrich, St. Louis, MO, USA) was used as a substrate to visualize positive staining. Finally, images were obtained by using an IX73 Olympus microscope equipped with a DP80 camera (Olympus Corp., Tokyo, Japan).

### 2.7. Western Blotting Assay

The gastric tissues were homogenised in RIPA Lysis Buffer and incubated on ice for 20 min, then the supernatants were collected by centrifugation at 13,000× *g* rpm for 20 min at 4 °C. A BCA protein assay kit was used to calculate protein concentrations (Nanjing JianCheng Bioengineering Institute, Nanjing, China). The total protein extract was separated on a 10% SDS-PAGE gel before being transferred to a PVDF membrane. After 120 min incubation in TBST with a blocking solution at room temperature, the membrane was incubated with a primary antibody at 4 °C overnight. After washing with TBST three times, the diluted secondary antibody was incubated at room temperature for 40 min. Protein bands were visualised using Labworks gel imaging equipment and software (Labworks LLC, Lehi, UT, USA) and an enhanced chemiluminescence detection kit (Thermo Fisher Scientific, Waltham, MA, USA).

### 2.8. Intestinal Microbiota Composition Analysis

A microbiota analysis was performed on caecal samples from each group and the number of samples satisfied the requirement of an intestinal flora diversity analysis. Microbial community genomic DNA from different caecal samples was extracted using an E.Z.N.A. ^®^Stool DNA Kit (D4015, Omega, Inc., Norcross, GA, USA) according to the manufacturer’s instructions. The bacterial small-subunit (16S) rRNA gene involving the V3-V4 region was amplified with slightly modified versions of the primers 338F and 806R. The PCR amplicons were purified with AMPure XT beads (Beckman Coulter Genomics, Danvers, MA, USA) and quantified by Qubit (Invitrogen, Carlsbad, CA, USA). The amplicon pools were prepared for sequencing and the quantity of the amplicon library was assessed using the Agilent 2100 Bioanalyzer (Agilent Technologies, Santa Clara, CA, USA) and the Library Quantification Kit for Illumina (Kapa Biosciences, Woburn, MA, USA), respectively. Samples were sequenced on an Illumina MiSeq PE300 platform (LC-Bio Technology Co., Ltd., Hangzhou, China) and analysed according to the manufacturer’s instructions. The richness and diversity of caecal microbiota were evaluated according to the method described in our previous study [22].

### 2.9. Statistical Analysis

Data were presented as the mean ± standard derivations (SDs) of three replicates. Analyses of significance were performed by the OriginPro Software Version 8.0 (OriginLab Corp., Northhampton, MA, USA). The results were analysed by a one-way analysis of variance (ANOVA), Hochberg test and two-sample *t* test. * *p* < 0.05 was considered statistically significant [25].

## 3. Results

### 3.1. Effect of SAFP Concentration on Intrinsic Viscosity

**Figure 1:**
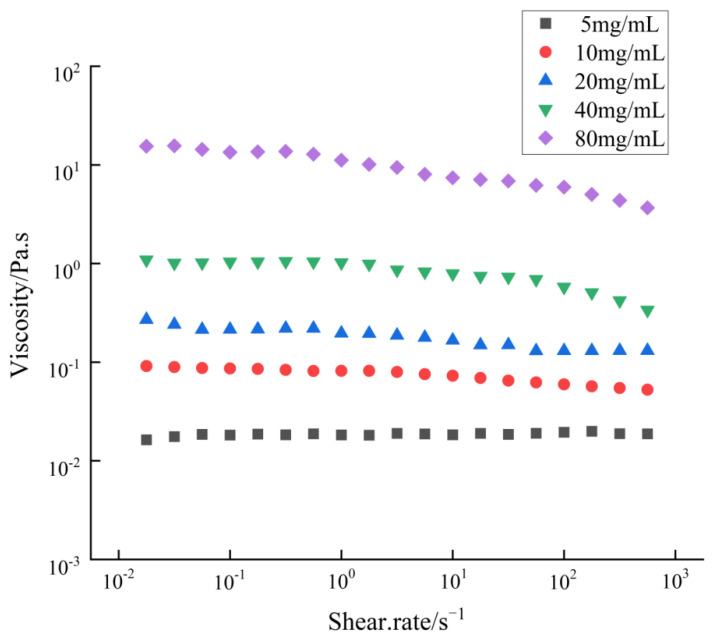
Variation of apparent viscosity of different concentration SAFP with shear rate.

As shown in Figure 1, the viscosity of the SAFP solution rose with the increased mass concentration from 5 to 80 mg/mL at a given shear rate. This may be due to the high polysaccharide content which induced the strong interactions between polysaccharide chains and resulted in an increased stable network-like structure or winding structure, and stronger shear forces were required to disrupt the network-like structure and release the fluid initially [26]. Furthermore, the apparent viscosity of the SAFP solution at various mass concentrations decreased with increasing shear rates in the range of 0.01–1000 s^−1^, which may be mainly ascribed to the shear-induced breakdown of an entangled polymer such as polysaccharides’ network and macromolecule interconnections during shearing, i.e., the intermolecular entanglement rate of disruption was greater than that of reformation, resulting in less intermolecular resistance to flow and lower apparent viscosity [27,28]. This is shear-thinning behavior, which indicated their typical non-Newtonian fluid nature. The apparent viscosity of the SAFP solution at 5 mg/mL concentration did not change significantly with the increase in the shear rate, indicating that the SAFP solution presented Newtonian fluid flow characteristics at this lower mass concentration.

### 3.2. Effect of Temperature on Intrinsic Viscosity

**Figure 2:**
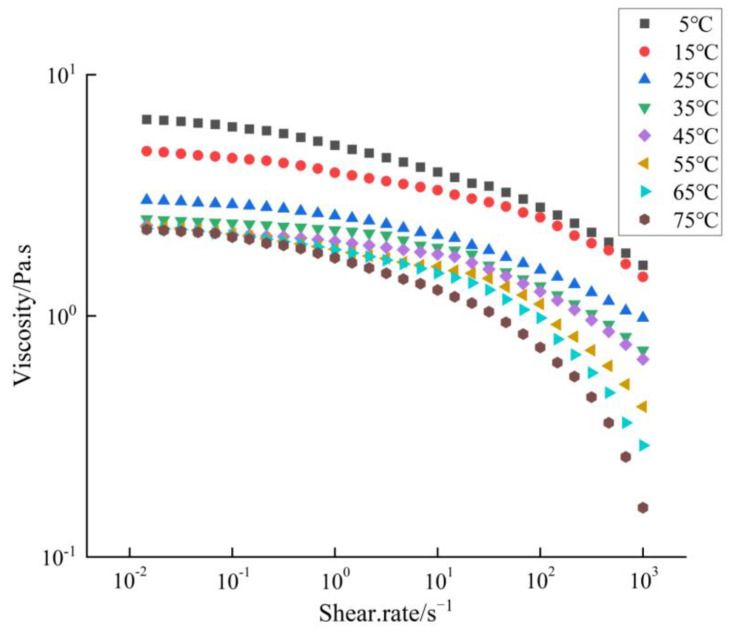
Variation of apparent viscosity of SAFP with temperature treatment.

As indicated in Figure 2, the viscosity of the SAFP solution at 20 mg/mL decreased when the temperature increased from 5 °C to 75 °C, which may be attributed to the intensified thermal motion of polysaccharide molecules with increasing temperature, which enlarged the intermolecular distance and weakened the intermolecular interactions [29,30]. This temperature-dependent result for the SAFP solution at 20 mg/mL was a kind of substantial non-Newtonian fluid characteristic [31].

### 3.3. SAFP Alleviates Oxidative Stress and Inflammation in Rats with Gastric Ulcer

**Figure 3:**
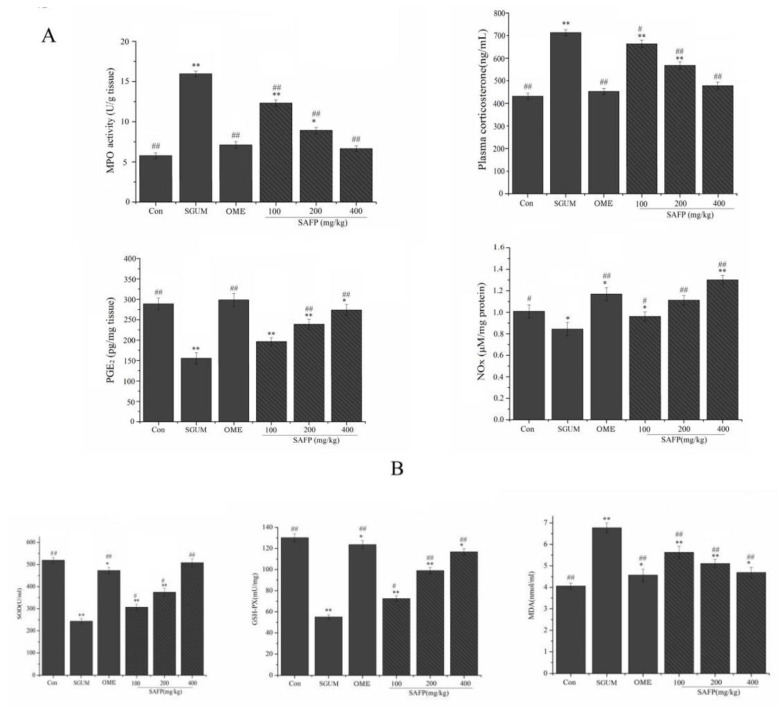

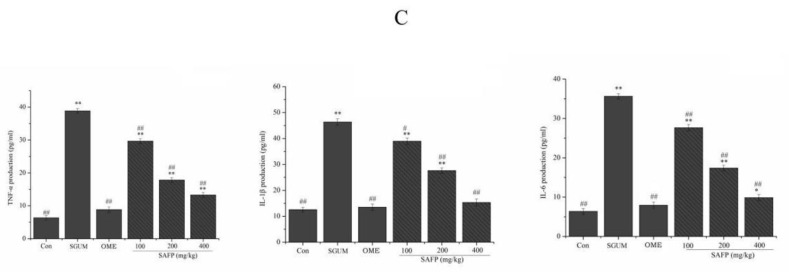
SAFP alleviates the inflammation and oxidative stress damage in SGUM rats. (**A**) Myeloperoxidase (MPO) activity; plasma corticosterone levels; the mean content of gastric Prostaglandin E2 (PGE2) and Nitrate/nitrite (NOx). (**B**) Effect of superoxide dismutase (SOD) activity; glutathione peroxidase (GSH-Px) activity; malondialdehyde (MDA) level. (**C**) The concentration of tumour necrosis factor-α (TNF-α), interleukin-1β (IL-1β) and interleukin-6 (IL-6) were determined by ELISA. Data are represented by mean ± SD (*n* = 10). * *p <* 0.05, ** *p <* 0.01, vs. control group. ^#^ *p <* 0.05, ^##^ *p <* 0.01, vs. SGUM group.

Myeloperoxidase (MPO) activity and plasma corticosterone levels were measured to evaluate the effect of SAFP on the release of glucocorticoids in stressed rats. As illustrated in Figure 3A, in the SGUM group, MPO activity and plasma corticosterone levels were dramatically upregulated when compared with the control group. However, SAFP pretreatment could reverse the increase in the plasma corticosterone concentration of rats induced by WIRS. The mean content of gastric PGE2 and NOx in gastric ulcer rats was significantly downregulated when compared with those in the control rats without stress. However, pretreatment with SAPF before the onset of WIRS could restore the depleted levels of PGE2 and NOx. The results in Figure 3B indicated that SOD and GSH-px levels were decreased in the SGUM group compared to the control group, while MDA was significantly increased. SAFP supplementation dramatically increased SOD and GSH-px levels but decreased MDA levels. As indicated in Figure 3C, in contrast to the control group, WIRS elicited a significant inflammatory response, strongly evidenced by the elevated levels of TNF-α, IL-1β and IL-6 (Figure 3C). However, compared with those in the SGUM group, the increases in these inflammatory factors were significantly reduced in SGUM rats pretreated with SAFP. These findings demonstrated that SAFP pretreatment could alleviate the oxidative stress-induced injury and ameliorated the inflammatory reaction in rats.

### 3.4. SAFP Alleviates WIRS-Induced Gastric Mucosal Injury in Rats

**Figure 4:**
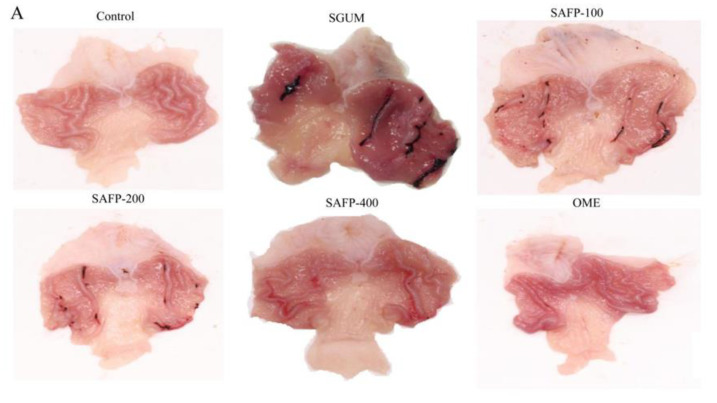

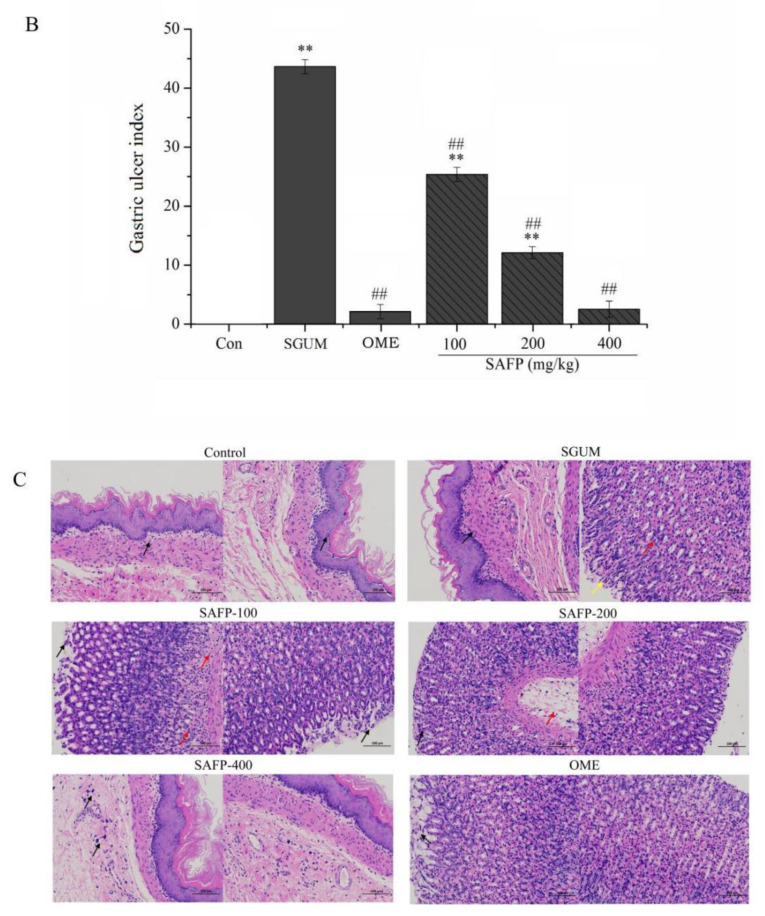
SAFP alleviates gastric mucosal injury. (**A**) The typical appearance of the gastric mucosa. (**B**) The gastric ulcer index (UI). (**C**) Pathological changes in lung tissues from each group of mice by H&E staining. Data are represented by mean ± SD (*n* = 10). ** *p <* 0.01, vs. control group. ^##^ *p <* 0.01, vs. SGUM group.

The typical appearance of the gastric mucosa is shown in Figure 4A. The surface of the gastric mucosa was smooth and intact without any macroscopic lesions in the control group. In the SGUM group, WIRS-induced haemorrhagic lesions in the gastric mucosa of stress rats presented as irregular dark red streaks of bleeding, diffuse gastric oedema, mucosal erythema and mucosal erosions, along with a high UI. Pretreatment with SAFP before the onset of WIRS significantly reduced the area of bleeding lesions and UI in rats compared with the SGUM group, even better than the positive OME group. As indicated in Figure 4B, in contrast with the control group, the gastric ulcer index (UI) was 43.65 ± 6.72 in the SGUM group (*p* < 0.05), while UI was significantly decreased (*p* < 0.05) dose-dependently in response to the SAFP treatment. The SAFPH (400 mg/kg) group exhibited the smallest UI (5.48 ± 5.85) and the highest percentage of ulcer inhibition (87.45%).

Next, we continued to investigate the effect of SAFP on pathological alternations in rats by H&E trichrome staining (Figure 4C). In the SGUM group, treatment with WIRS remarkably resulted in the severe damage to the gastric mucosa epithelium and submucosal oedema, inflammation infiltration and gastric gland epithelial cell necrosis, a considerable loss of gastric mucosal epithelial cells. Notably, rats pretreated with SAFP significantly ameliorated the mucosal damage and haemorrhagic lesions as well as the reduced infiltration of neutrophils into the surface epithelium. These histological appearances are essentially the same as those that were pretreated with Omeprazole, serving as the positive drug control.

### 3.5. SAFP Attenuates Stress Gastric Ulcer via Reducing the Levels of Marker Proteins

**Figure 5:**
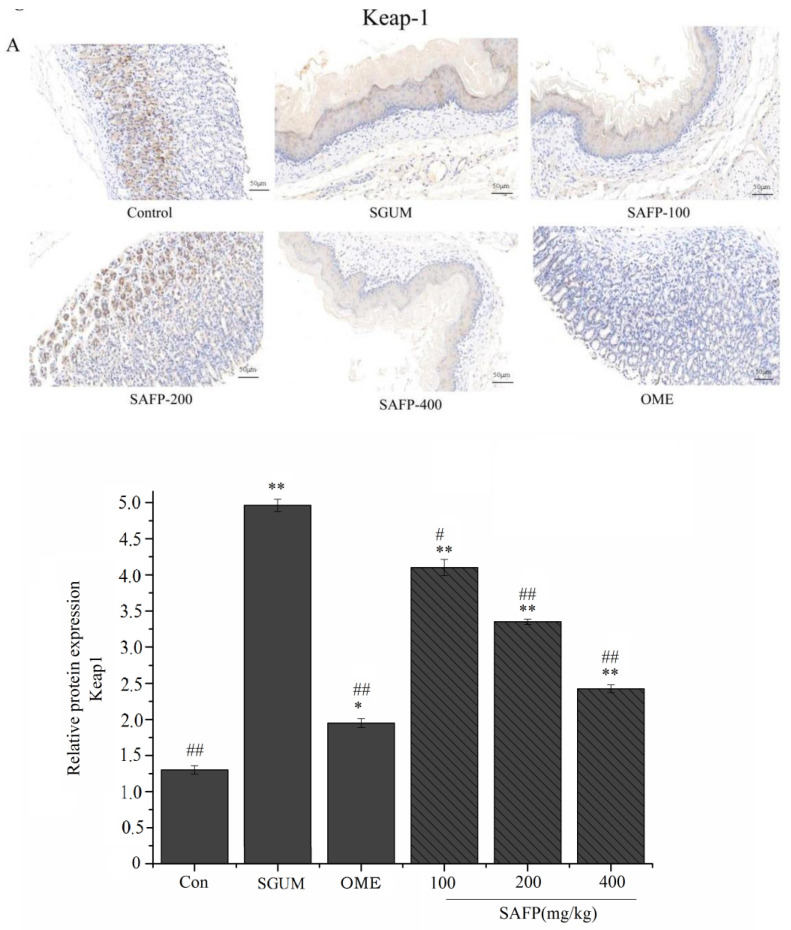

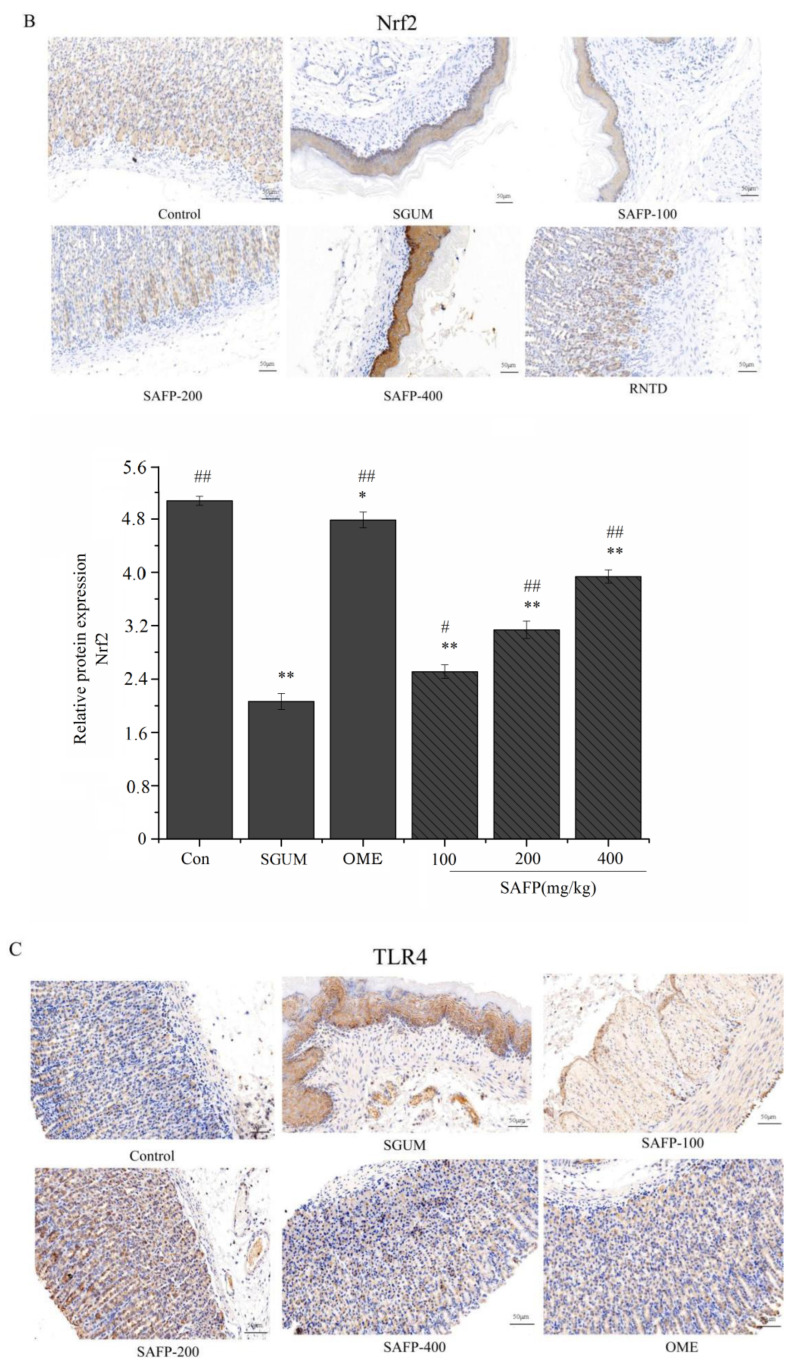

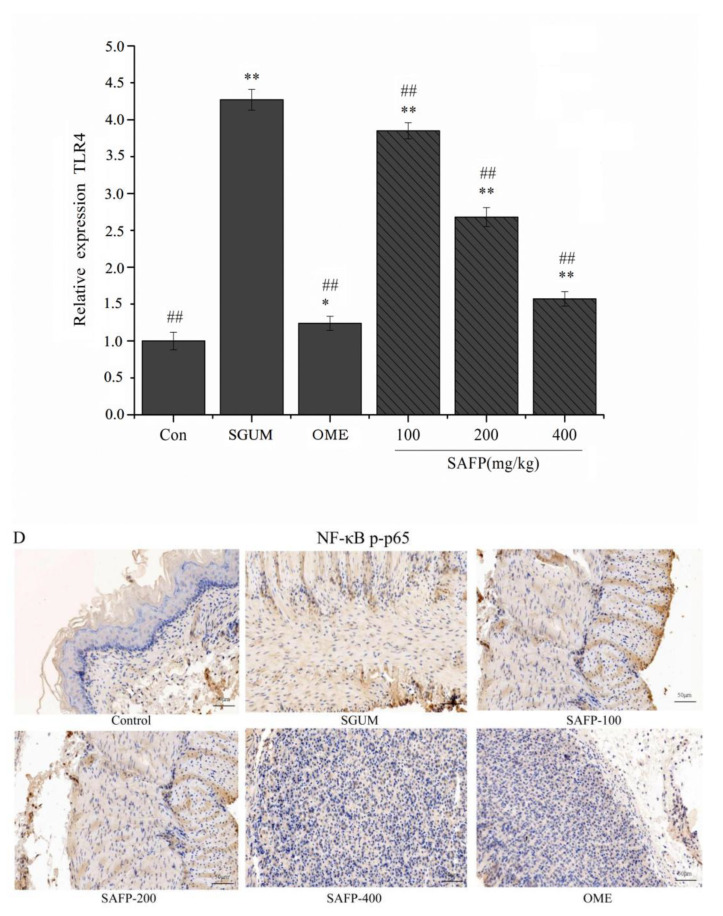

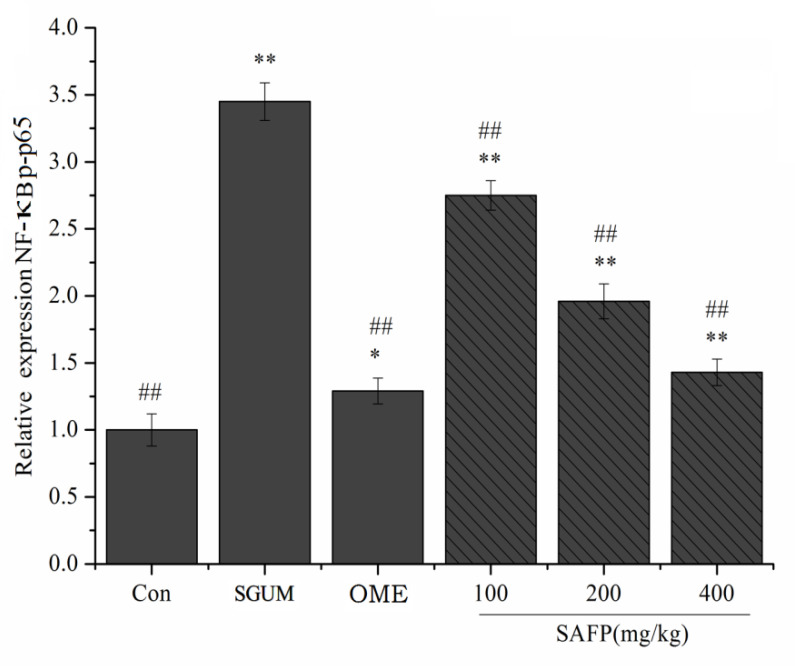
Immunohistochemical assay. (**A**) Expression of Keap-1 by immunohistochemical staining and statistical analysis of Keap-1. (**B**) Expression of Nrf2 by immunohistochemical staining and statistical analysis of Nrf2. (**C**) Expression of TLR4 by immunohistochemical staining and statistical analysis of TLR4. (**D**) Expression of NF-κBp-p65 by immunohistochemical staining and statistical analysis of NF-κBp-p65. Data are represented by mean ± SD (*n* = 10). * *p* < 0.05, ** *p* < 0.01 vs. control group. ^#^ *p* < 0.05, ^##^ *p* < 0.01, vs. SGUM group.

**Figure 6:**
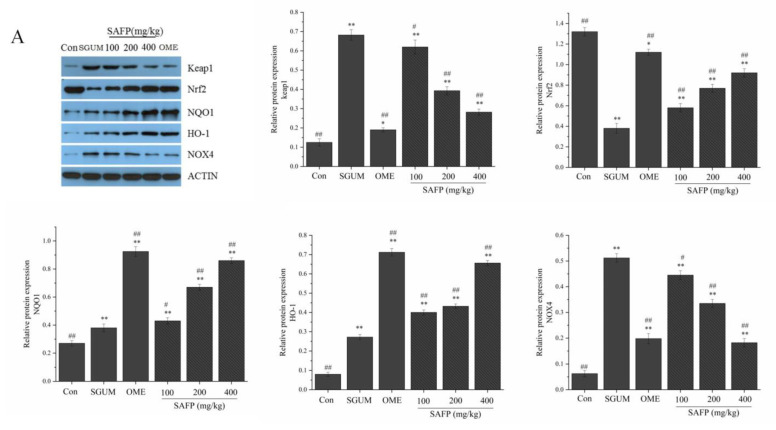

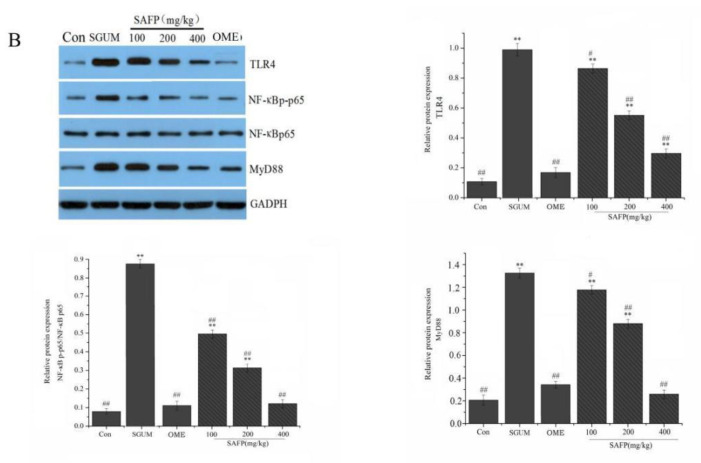
SAFP protects against gastric mucosal injury through Nrf2 and toll-like receptor 4 (TLR4) signaling pathways. (**A**) Western blot assay of Keap-1, Nrf2, NQO1, *HO-1* and NOX4 expression; quantification of relative protein expression quantity of Keap-1, Nrf2, NQO1, *HO-1* and NOX4. (**B**) Western blot assay of TLR4, NF-kB p-p65, NF-kB p65 and MyD88 expression; quantification of relative protein expression quantity of TLR4, NF-kB p-p65, NF-kB p65 and MyD88. Data are represented by mean ± SD (*n* = 10). * *p <* 0.05, ** *p <* 0.01, vs. control group. ^#^ *p <* 0.05, ^##^ *p <* 0.01, vs. SGUM group.

The immunohistochemical staining patterns of marker proteins were studied to determine whether SAFP attenuates inflammation and stress ulcers. The upregulated expression of Keap-l, TLR4 and NF-κB p-p65 (Figure 5A,C,D), and downregulated expression of Nrf2 (Figure 5B) in the gastric tissue were observed via immunoexpression in the SGUM group, which were reversed remarkably when pretreated with SAFP and Omeprazole. Furthermore, the protein levels were assessed by Western blotting in gastric tissues, and a comparable trend appears in Figure 6.

### 3.6. SAFP Alleviates Gastric Mucosal Injury via the TLR4 and Nrf2 Signaling Pathways

The molecular mechanisms underlying the alleviating effect of SAFP on WIRS-induced gastric mucosal injury was investigated by a Western blotting assay. Figure 6A showed that the expressions of Keap-1, NQO-1, *HO-1* and NOX4 were markedly increased, while Nrf2 was decreased in the gastric tissues of the SGUM group (*p* < 0.01). As indicated in Figure 6B, in contrast to the control group, WIRS had significantly upregulated the protein expression of TLR4, NF-κB p-p65/NF-κBp65 and MyD88. Notably, the supplementation of SAFP and Omeprazole upregulated the protein expressions of Nrf2, NQO-1 and *HO-1*, and downregulated the protein expressions of Keap-1, NOX4, TLR4, NF-κB p-p65/NF-κBp65 and MyD88 when compared with the SGUM group. The effect of SAFP was comparable to Omeprazole when the dose was 400 mg/kg. Overall, these results provided evidence that SAFP could play a dominant role in protective effects on gastric mucosal injury by activating Nrf2 pathways and inhibiting the TLR4 pathway.

### 3.7. SAFP Modulated WIRS-Induced Gut Microbiota Dysbiosis

**Figure 7:**
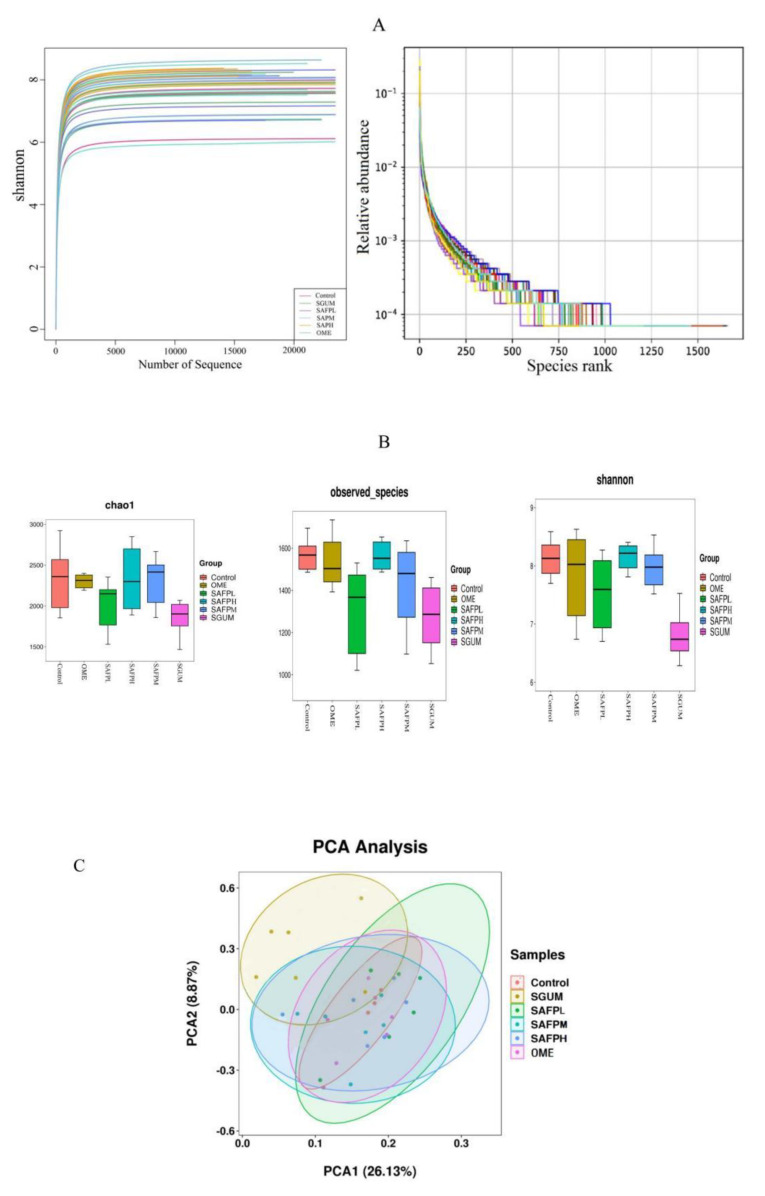

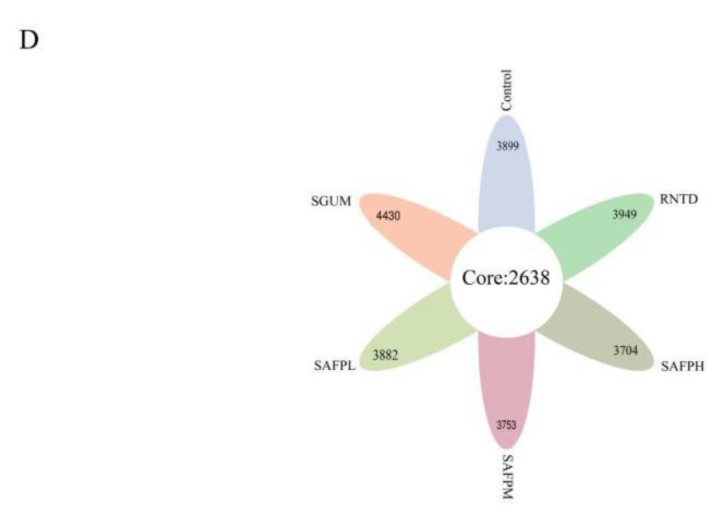
Effects of SAFP on the richness and diversity of caecal microbiota (*n* = 5). (**A**) Shannon index curve and rank abundance curve. (**B**) Operational taxonomic unit (OTU) richness and diversity indices. (**C**) Principal coordinate analysis score plot of gut microbiota. (**D**) Petal diagram presenting the OTUs between different groups. SAFPL group represents SAFP low-dose group (100 mg/kg BW); SAFPM group represents SAFP middle-dose group (200 mg/kg BW); SAFPH group represents SAFP high-dose group (400 mg/kg BW).

To assess the effect of SAPF on the intestinal microbiota in SGUM rats, we analysed the diversity and composition of microbes in faeces. Alpha diversity was assessed by the sparsity curve (Shannon index curve) and rank abundance curves which indicated that sequencing depth was sufficient to reflect the microbial diversity of the community in each sample (Figure 7A). An alpha diversity analysis represented as four indexes, Chao, Shannon and Simpson, was used to evaluate microbial richness and diversity (Figure 7B). The SAFP pretreatment resulted in the increased levels of Chao1 and Shannon indexes, indicating that SAPF can improve the richness and diversity of intestinal bacteria when compared with that in the SGUM group, with a similar effect to that of Omeprazole.

A principal coordinate analysis (PCoA) was performed to calculate beta diversity, which indicated an obvious clustering of the microbiome composition for each treatment group (Figure 7C). The result indicated an obvious difference between the microbiota composition of SGUM and SAFP groups, which suggested that SAFP might increase microbial diversity and stabilise the microbial community in rats. A petal diagram was used to explore the OTUs; a total of 26,255 OTUs were detected, indicating species’ numbers, of which 1638 were shared among groups. Furthermore, 4199, 4430 and 4049 unique OTUs were observed in the control, SGUM and OME groups (Figure 7D).

**Figure 8:**
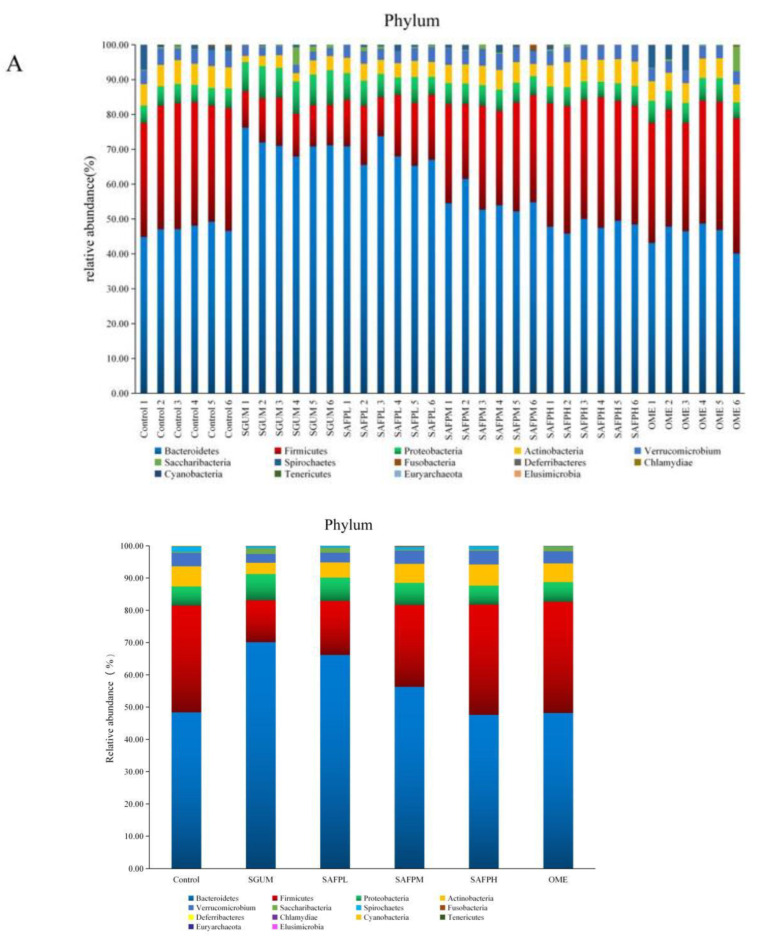

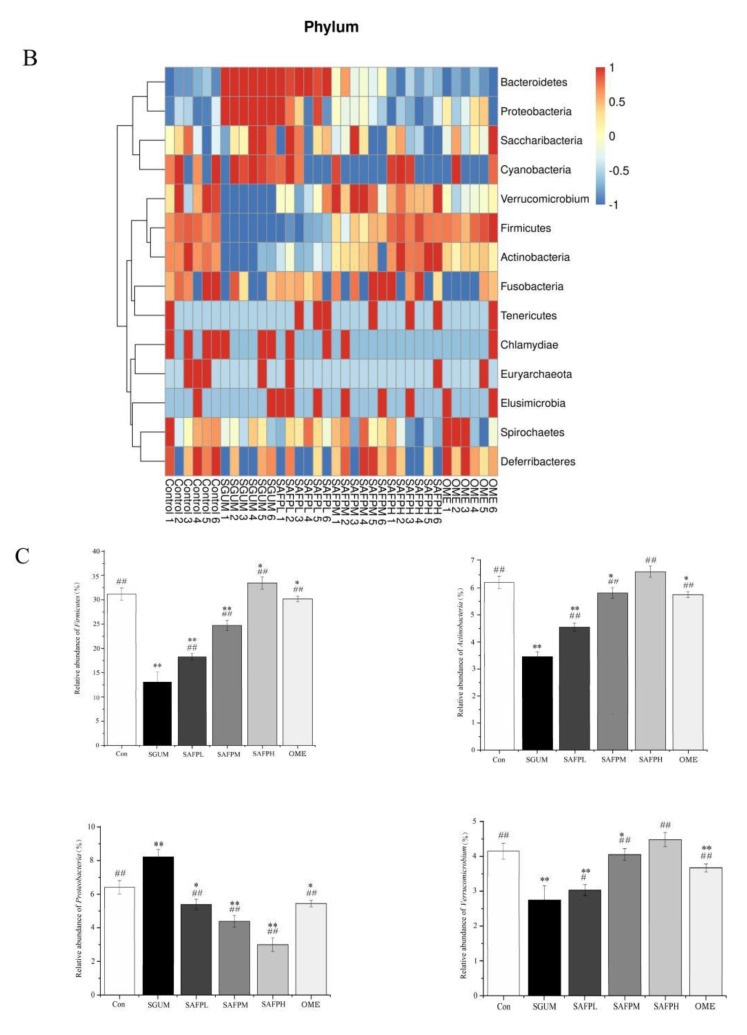

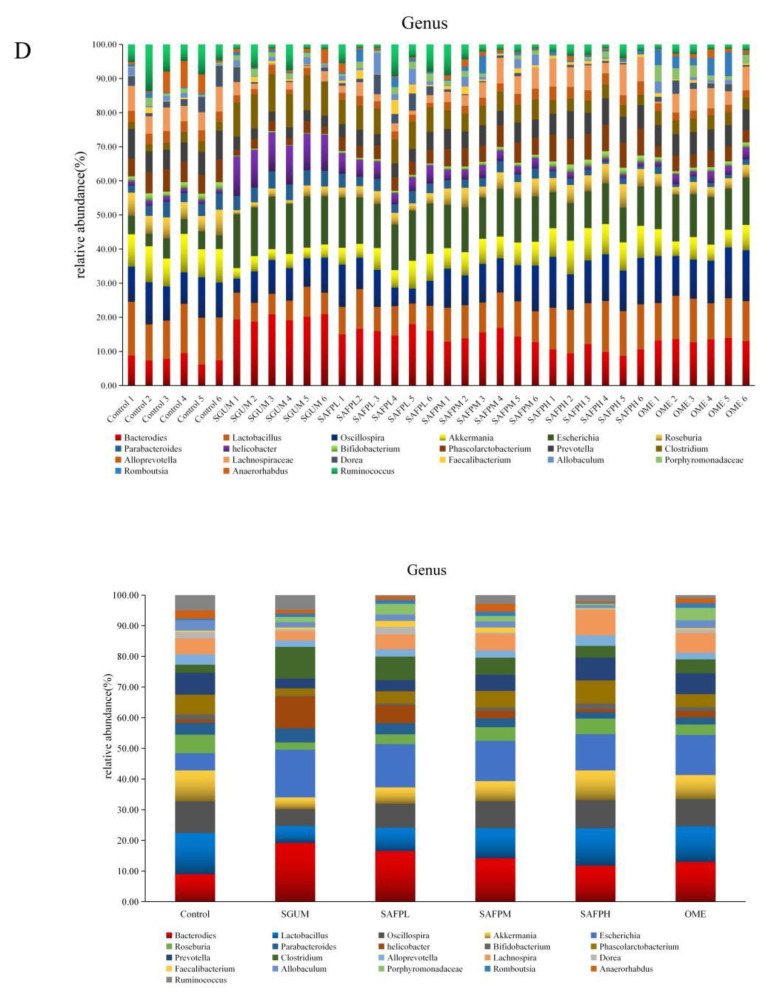

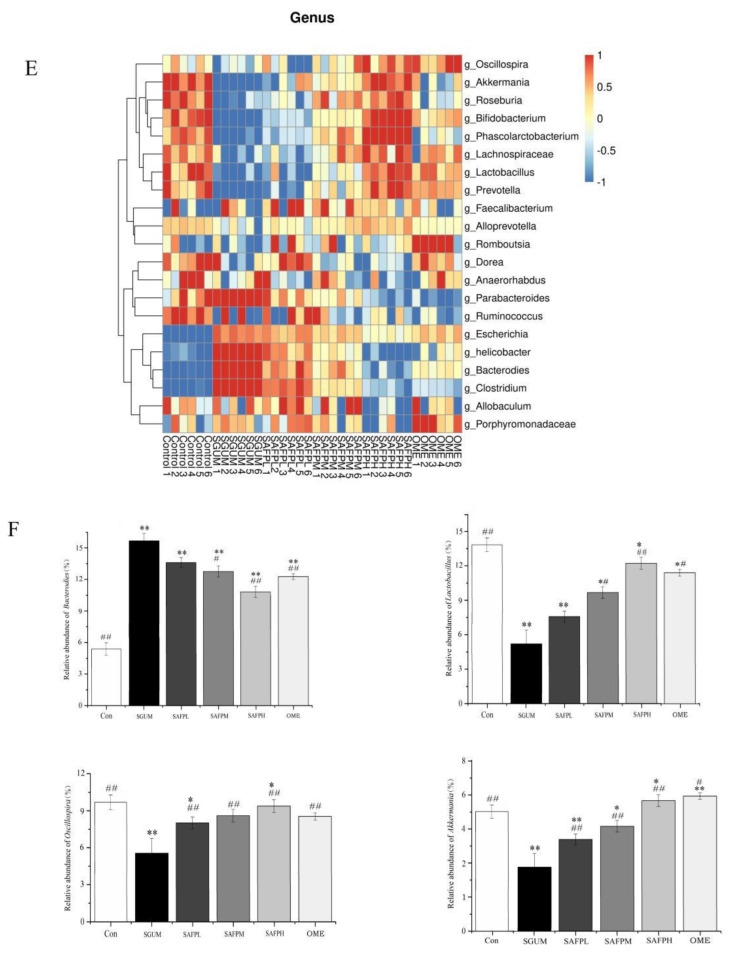

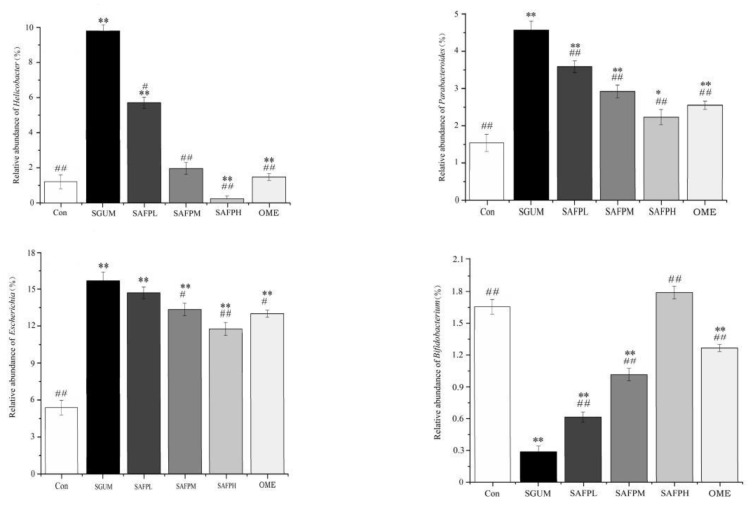
SAFP modulated the composition and structure of caecal microbiota (*n* = 5). (**A**) Relative abundance of bacteria at the phylum level. (**B**) Relative abundances of predominant phylum by heatmap analysis. (**C**) Effects of SAFP on the relative abundance of Firmicutes, Actinobacteria, Proteobacteria and Verrucomicrobia. (**D**) Relative abundance of bacteria at the genus level. (**E**) Relative abundances of predominant genus by heatmap analysis. (**F**) Effects of SAFP on the relative abundance of *Bacterodies, Lactobacillus, Oscillospira, Akkermania, Helicobacter*, *Parbacterodies, Escherichia and Bifidobacterium.* * *p <* 0.05, ** *p <* 0.01, vs. control group. ^#^ *p <* 0.05, ^##^ *p <* 0.01, vs. SGUM group. SAFPL group represents SAPF low-dose group (100 mg/kg BW); SAFPM group represents SAFP middle-dose group (200 mg/kg BW); SAFPH group represents SAFP high-dose group (400 mg/kg BW).

The predominant taxonomic profiling and heatmapping was used to further analyse the abundance of predominant taxa at the phylum level in each group. Then, as indicated in Figure 8A–C, the microbial community structure was dominated by *Firmicutes*, *Actinobacteria*, *Proteobacteria* and *Verrucomicrobia*, which accounted for more than 90% of all abundance. WIRS treatment increased the relative abundances of Proteobacteria by 66.78%. However, WIRS treatment decreased the relative abundances of Firmicutes from 34.10% to 13.00%, *Actinobacteria* from 6.23% to 3.47% and Verrucomicrobia from 4.23% to 2.65% (*p* < 0.01). In contrast, SAFP and Omeprazole supplementation could significantly downregulate the abundances of Proteobacteria while upregulating the abundances of *Firmicutes*, *Actinobacteria* and *Verrucomicrobia*, which returned to normal levels (*p* < 0.01). Furthermore, the intestinal microbiota was analysed at the genus level (Figure 8D–F). *Bacteroides*, *Lactobacillus*, *Oscillospira*, *Escherichia*, *Helicobacter* and *Akkermansia* were dominant flora at the genus level. In contrast to the control group, the relative abundance of *Oscillospira*, *Lactobacillus*, *Akkermansia* and *Bifidobacterium* were dramatically downregulated in the SGUM group, which were significant upregulated by SAFP and Omeprazole. Furthermore, in contrast to the SGUM group, being pretreated with SAFP led to a marked reduction in the relative abundance of *Bacteroides*, *Helicobacter*, *Escherichia* and *Parabacteroides*. The results suggested that microbiome phenotypes were similar between the control and SAFP-treated groups. Thus, SAPF could modulate the intestinal microbiota dysbiosis by upregulating the beneficial bacterium and downregulating pathogenic bacteria or conditioned pathogens. The effect of SAFP was comparable to, or even better than, Omeprazole when the dose was 400 mg/kg.

## 4. Discussion

The occurrence and development of a stress gastric ulcer is closely related with the oxidative stress of cells. The increased sensitivity of the hypothalamic–pituitary–adrenal axis after stress causes an increased secretion of gastric acid, pepsin and gastrin. Meanwhile, the release of cortisol and catecholamine can induce the production of oxygen free radicals, further causing the formation of lipid peroxides in the cell membrane and, consequently, oxidative stress [20]. It is well known that polysaccharides from plants, fungi and marine organisms are recognised as an antioxidant supplement with potential value in the treatment or prevention of oxidative stress damage and have gained widespread interest [32,33,34,35].

As a transcription factor, Nrf2 is coupled with the cytoplasmic chaperone protein Keap-l and anchored to the cytoplasm in the physiological state. This process causes Nrf2 to go into a relatively inactivated state. This process results in the transcriptional activation of phase II enzymes/antioxidant genes mediated by ARE such as *HO-1*, NQO1 and NOX4, increasing the GSH-px and SOD levels to maintain the intracellular redox balance and remove the excess oxygen free radicals, thereby playing an anti-stress role [36]. Our results suggested that WIRS treatment may lead to the dissociation of Nrf2 from the dimer and transfer into the nucleus in the cytoplasm of the gastric fundus cells, thus playing an anti-stress role. *HO-1* is an inducible heme oxygenase that could reduce the sensitivity of gastrointestinal cells to oxidant damages. Studies have confirmed that *HO-1* was considered as a stress protein and most notably associated with the protection of the gastrointestinal tract from anti-inflammatory and anti-tissue damage, and antioxidation [37]. It also protects the cell mucosa by regulating the gastrointestinal movement under stress and disease conditions. The Nrf2 signaling pathway is critical in the processes of cellular antioxidant damage and inflammatory damage [38]. Studies have reported that *Mangiferin* mediated its gastroprotective effect in an ischaemia/reperfused rat model partly by inducing the expressions of Nrf2, *HO-1* and PPAR-γ and downregulating the expression of NF-κB [39]. The activation of Nrf2 mitigated the gastric ulcers in ethanol-induced gastric ulcers in rats and protected the damage of gastric mucosa in NSAID-induced gastric ulcers by decreasing the production of ROS [40].

Patients with a gastrointestinal disease have unbalanced intestinal flora, mucosal barrier function and altered permeability because of the translocation and entry of the Gram-negative bacteria in the lymphatic and blood systems. The endotoxin lipopolysaccharide (LPS) of Gram-negative bacteria stimulates the innate immune system and binds to the toll-like receptor 4 (TLR4), further leading to signal transduction into the cells. Increasing evidence in water immersion and restraint stress (WIRS) mice has indicated that TLR4 stimulation leads to an inflammatory response, the production of inflammatory cytokines and the chemokine-mediated recruitment of acute inflammatory cells [41].

As a key transcription factor, the nuclear factor-κB (NF-κB) plays an important role in regulating cell apoptosis and stress response. NF-κB is sequestered in the cytoplasm and bound by its inhibitor IκBα in resting cells. Once stimulated by LPS, IκBα molecules accelerate their degradation and release NF-κB in the cytoplasm, which translocates to the nucleus and triggers the transcription of specific target genes including TNF-α, IL-1β and IL-6, thereby promoting the release of various inflammatory cytokines and resulting in an inflammatory reaction [42]. Studies have shown that chronic inflammation plays a crucial role in the occurrence and progression of gastric ulcers. The activation of the TLR4 pathway is a major factor in promoting the inflammatory response and gastric mucosa injury [23]. Previous studies have demonstrated that TLR4 and Nrf2 pathways maintain a redox homeostasis and further modulate the key redox modulators by individually affecting various signaling cascades to interact with each other [43]. The activation of Nrf2 pathways alleviates the gastric mucosa injury by promoting antioxidant defences and suppressing the activation of the NF-κB pathway. In this study, we postulated that SAFP exhibited an anti-inflammatory activity that can be attributed to the suppression of the NF-κB signaling cascade and release of inflammatory cytokines. In summary, WIRS activated the TLR4 signaling pathway and led to a serious inflammatory response, whereas SAFP exerted anti-inflammatory action and protective effects on WIRS-induced gastric mucosa injury by activating Nrf2 pathways and suppressing the TLR4 pathway.

Gut microbiota play an essential role in initiating or maintaining intestinal immunity in inflammatory responses by providing antigens or other stimulators, which are closely related to the occurrence of gastrointestinal disease. The homeostasis of the gut microbiota is a key factor which affect gastrointestinal health. Gastrointestinal diseases interfere with the homeostasis of intestinal microbiota, thereby leading to gut microbiota dysbiosis. The richness and diversity of gut microbiota were found to be significantly different in SGUM rats compared to normal rats in our study. However, the SAFP and OME groups exhibited the healthy microbiota community, among which, the SAFPH group (400 mg/kg) showed the best regulation effect on gut microbiota. Some polysaccharides, such as *Bupleurum* polysaccharides, may act as prebiotics to prevent changes in the intestinal microbial communities [16]. The abundance of *Firmicutes* was associated with energy absorption and higher levels of inflammation [44]. Gastrointestinal inflammation can lead to an increased consumption of energy. Therefore, the body absorbs energy by increasing the presence of *Firmicutes* to eliminate inflammation in the digestive tract after being pretreated with SAFP. *Actinobacteria* have anti-tumour effects that reduce the activity of harmful bacterium in the intestinal tract and can also maintain the relative stability of species and quantity of intestinal flora [45]. After the upregulation of inflammatory cytokines, there were decreased quantities of *Firmicutes* and *Actinobacteria* and increased quantities of *Bacteroidetes* in SGUM rats. This change in quantities was markedly reversed by SAPF intervention after the downregulation of inflammatory cytokines. The relative abundance of harmful bacterium, including *Proteobacteria*, that could promote the development of inflammation and stimulate the formation of ulcer foci, was enhanced significantly in SGUM rats. However, the abundance was reduced by SAFP and Omeprazole supplementation. Probiotics such as *lactobacillus* and *Bifidobacterium* are known to be able to regulate the immune function of the gastrointestinal tract and inhibit NF-κB activation [46], prevent exogenous stimuli and the invasion of harmful bacteria, decrease intestinal permeability and contribute to the regeneration of vascular endothelial factors, thereby promoting the healing of gastric mucosa and assisting in the treatment of ulcers [37,38,39,40,41,42,43,44,45,46,47,48,49]. In addition, it has been found that probiotics such as *Lactobacillus plantarum* ZDY2013 and *Bifidobacterium bifidum* WBIN03 can regulate the secretion of antioxidant enzymes, SOD1, GPX2 and CAT, by activating the Nrf2 pathway, while achieving the effect of alleviating colitis [50]. *Akkermansia* contributes to maintaining a healthy digestive tract and reducing the risks of obesity, diabetes, inflammation and other diseases.

Moreover, previous reports have indicated that the elevated levels of Akkermansia abundance were positively related with intestinal integrity and intestinal tight junctions [51], whereas the decrease in abundance led to the reduced resistance of gastrointestinal mucosa to external invasion. In our study, the abundance of Bacteroides, Akkermansia and lactobacillus were significantly enhanced by SAFP and Omeprazole supplementation in the SGUM group. The most common causes of peptic ulcers are the abnormal secretion of gastric acid and *Helicobacter* infection. *Bacteroides*, *Parabacteroides* and *Helicobacter* are commonly considered as pathogenic bacteria or conditioned pathogens in the gastrointestinal tract. For example, the elevated levels of *Bacteroides* and *Parabacteroides* can cause colitis in mice, *Helicobacter pylori* infection can induce peptic ulcers or gastric cancers and *Escherichia* in vivo is conducive to the development of intestinal inflammation [52,53,54]. The basic condition of *Helicobacter* colonisation in the human body is the disorder of gastrointestinal microbiota, which makes it the dominant bacterium in the gastrointestinal tract of SGUM rats [55]. The main metabolic end products of *Parabacteroides* are acetic acid and succinic acid, which help in relieving inflammation. We observed that the quantities of *Bacteroides*, *Parabacteroides* and *Helicobacter*, which are closely associated with the severity of stress gastric ulcer, increased significantly in SGUM rats and were decreased with SAFP and Omeprazole treatment. *Oscillospira* could play an important anti-inflammatory role when there is an inflammatory reaction in the digestive tract [56]. Butyrate, as one of the components of short-chain fatty acids, is thought to be a key energy source that aids in the maintenance of the intestinal barrier while also protecting the host from potential pathogens into the gastrointestinal cavity [11]. Patients with peptic ulcers had reduced numbers of butyrate-producing bacteria such Roseburia and other bacteria, according to previous studies [57]. Surprisingly, the abundance levels of *Oscillospira* and *Roseburia* were significantly increased when pretreated with SAFP. Consequently, we observed and analysed the correlation between inflammatory cytokines and microbiota. SAFP had beneficial effects on the gut microbiota homeostasis by improving the diversity of the intestinal microbial community, and improving the relative abundance and proliferation capacity of beneficial bacterium, while reducing the proliferation of pathogenic bacteria or conditioned pathogens.

## 5. Conclusions

In summary, our research is the first attempt to evaluate the protective effects of polysaccharides from *Sarcodon aspratus* on WIRS-induced gastric ulcers in rats. The results demonstrated that polysaccharide SAFP could relieve the gastric mucosal injury and haemorrhagic lesions in rats by alleviating the inflammatory response, via the activation of the Nrf2 signaling pathway and suppression of the TLR4 signaling pathway. In addition, the effects of SAFP might be associated with its modulatory effects on gut microbiota dysbiosis. Therefore, our findings demonstrated that SAFP might be explored as a natural gastric mucosal protective agent and potential health supplement in the prophylactic effects of gastric ulcer and related complications in food and pharmaceutical industries, which may improve our understanding of *Sarcodon aspratus* polysaccharides as functional food.

## Data Availability

Data is contained within the article.

## Abbreviations

Abbreviation	Full name
GU	Gastric ulcer
NSAIDs	Nonsteroidal anti-inflammatory drugs
IBD	Inflammatory bowel disease
SAFP	Sarcodon aspratus fruiting polysaccharide
WIRS	Water immersion and restraint stress
SD	Sprague-Dawley
SGUM	Stress gastric ulcer model
OME	Omeprazole
SAFPL	SAFP low-dose
SAFPM	SAFP middle-dose
SAFPH	SAFP high-dose
UI	Ulcer index
PGE2	Prostaglandin E2
MPO	Myeloperoxidase
PCoA	Principal coordinate analysis
TLR4	Toll-like receptor 4
NF-κB	Nuclear factor-κB
